# Glyceraldehyde-3-phosphate dehydrogenase from *Eimeria acervulina* modulates the functions of chicken dendritic cells to boost Th1 type immune response and stimulates autologous CD4^+^ T cells differentiation in-vitro

**DOI:** 10.1186/s13567-020-00864-z

**Published:** 2020-11-17

**Authors:** Shakeel Ahmed Lakho, Muhammad Haseeb, Jianmei Huang, Zhang Yang, Muhammad Waqqas Hasan, Muhammad Tahir Aleem, Muhammad Ali-ul-Husnain Naqvi, Muhammad Ali Memon, XiaoKai Song, RuoFeng Yan, Lixin Xu, XiangRui Li

**Affiliations:** MOE Joint International Research Laboratory of Animal Health and Food Safety, College of Veterinary Medicine, Nanjing Agricultural University, Nanjing, Jiangsu People’s Republic of China

**Keywords:** *Eimeria* species, glyceraldehyde-3-phosphate dehydrogenase, chicken dendritic cells, CD4^+^ T cells, cytokines, toll-like receptors, coccidiosis

## Abstract

Dendritic cells (DCs) play a pivotal role to amplify antigen-specific immune responses. Antigens that sensitize T cells via antigen-presentation by DCs could enhance the capacity of host immunity to fight infections. In this study, we tested the immunogenic profiles of chicken DCs towards Glyceraldehyde-3-phosphate dehydrogenase from *Eimeria acervulina* (EaGAPDH). Immunoblot analysis showed that recombinant EaGAPDH (rEaGAPDH) protein was successfully recognized by rat sera generated against rEaGAPDH. Interaction and internalisation of rEaGAPDH by chicken splenic-derived DCs (chSPDCs) was confirmed by immunofluorescence analysis. Flow cytometry revealed that chSPDCs upregulated MHCII, CD1.1, CD11c, CD80, and CD86 cell-surface markers. Moreover, mRNA expressions of DC maturation biomarkers (CCL5, CCR7, and CD83) and TLR signalling genes (TLR15 and MyD88) were also upregulated whereas those of Wnt signalling were non-significant compared to negative controls. rEaGAPDH treatment induced IL-12 and IFN-γ secretion in chSPDCs but had no effect on IL-10 and TGF-β. Likewise, DC-T cell co-culture promoted IFN-γ secretion and the level of IL-4 was unaffected. Proliferation of T cells and their differentiation into CD3^+^/CD4^+^ T cells were triggered in chSPDCs-T cells co-culture system. Taken together, rEaGAPDH could promote Th1 polarization by activating both host DCs and T cells and sheds new light on the role of this important molecule which might contribute to the development of new DCs-based immunotherapeutic strategies against coccidiosis.

## Introduction

Chicken coccidiosis is a complex intestinal disease caused by intracellular protozoan parasites of the genus *Eimeria*. This disease results in diarrhoea, dehydration, loss of weight, and mortality in susceptible birds [2, 9]. It has, therefore, a major influence on commercial poultry settings. The common control methods, including prophylactic medication and vaccination with live or attenuated parasites, have serious limitations [39]. For instance, prophylactic medications are commonly encountered with drug-resistance by *Eimeria* parasites. In addition, the attenuated parasite vaccines are at risk of regaining their pathogenicity in immunocompromised birds. Moreover, the protective immunity is mostly *Eimeria’s* species-specific resulting in limited immunological cross-reactivity of antigens among *Eimeria* species [6, 9, 39]. Therefore, novel alternate approaches are required for the successful control of this disease. Particularly, it is crucial to design next-generation vaccines comprising of protective antigens that provide effective immune responses against all relevant species of parasite, *Eimeria.*

Dendritic cells (DCs) are the most efficient antigen-presenting cells that form a critical link between innate and adaptive immunity through interactions with T lymphocytes [1]. In the context of intracellular parasites such as *Eimeria*, the main protective immunity is generally T cells dependent [4, 31], however, the crucial step in the activation of naïve T cells into effector cells is determined by the maturation status of DCs [1]. Therefore, an in-depth understanding of the immunogenic profile of host DCs in response to parasite-derived antigens could help to identify the novel candidate antigens for vaccination against chicken coccidiosis.

Glyceraldehyde-3-phosphate dehydrogenase (GAPDH), one of the important enzymes in glycolysis, has various functions in eukaryotic pathogens, such as cell signalling, controlling gene expression, and interaction with other proteins [35]. This molecule is shared across the developmental stage of *Eimeria* parasites [23] and plays a pivotal role during the infection process of pathogens [38]. GAPDH is considered as an attractive target for drugs against protozoan parasites since these parasites lack a functional citric acid cycle and are dependent solely on glycolysis for energy requirements [42]. An earlier study indicated that GAPDH is one of the common molecules among the most economically important *Eimeria* species of poultry viz. *Eimeria tenella* (*E. tenella*) *Eimeria maxima* (*E. maxima*), and *Eimeria acervulina* (*E. acervulina*) [28]. More recently, immunoproteomic and mass spectrometric analysis by Liu et al. demonstrated that GAPDH protein is one of the immunologically shared antigens in *E. tenella*, *E. acervulina,* and *E. necatrix* [27]. Moreover, as a common antigen of *Eimeria*, GAPDH has been shown to protect chickens against *E. tenella*, *E. acervulina*, *E. maxima*, and mixed infection of the three *Eimeria* species [41]. However, the immunomodulatory role of this molecule in the host DCs is not yet explored.

In the present study, we aimed to assess the role of GAPDH from *E. acervulina* in the phenotypical and functional activation of chicken splenic-derived DCs (chSPDCs) and DC-dependent priming of T cells that potentially contribute to the development of novel therapeutic strategies for controlling coccidiosis.

## Materials and methods

### Animals

Hy-Line 1-day-old chickens were reared in a pathogen-free environment. Birds were supplied with standard coccidiostat-free feed and water ad-libitum throughout the study. Thirty-day-old Sprague Dawley rats (Qinglong Mountain Animal Breeding Farm, China) were also obtained and maintained under sterile environment.

### Preparation of rEaGAPDH recombinant protein

Constructed recombinant plasmid pET-32a-EaGAPDH (EaGAPDH accession ID: XM_013395851.1) was provided by the Laboratory of parasitology and molecular immunology, Nanjing Agricultural University (Nanjing, China). Bacterial strain**,** BL21 (DE3), was used for the transformation of recombinant plasmid in Luria–Bertani medium with ampicillin (100 µg/mL), and 1 mM Isopropyl-β-d-thiogalactopyranoside (Sigma-Aldrich) was used to induce protein expression. After 6 h of culture, cells were harvested. Thereafter, the cell suspension was sonicated for 30 min. The recombinant EaGAPDH (rEaGAPDH) protein was purified with the supernatant (soluble) of bacterial lysates using His TrapTM FF column (GE Healthcare, USA). The protein expression was analyzed by 12% SDS-PAGE. The Pierce™ BCA protein assay kit (Thermo Scientific, USA) was utilized to measure the concentration of purified protein. His-Tag protein (pET-32a empty protein) was also purified using the same protocol as defined for rEaGAPDH and used as negative vector control and stored at – 80 °C for downstream assays.

### Immunoblot analysis

Anti-rEaGAPDH antibodies were generated in SD rats as described previously [22]. After electrophoresis of rEaGAPDH on 12% SDS-PAGE, the purified proteins were transblotted to PVDF transfer membrane (Millipore Corporation, Billerica, MA, USA). Subsequently, 5% skimmed dry milk in Tris-buffered saline (TBS) with 0.5% Tween-20 (TBST) was used to block the membrane. The membranes were subjected to incubation with primary antibodies (anti-rEaGAPDH generated in SD rat) diluted to 1:100 with TBST at 37 °C for 2 h. After following standard washing procedures, the membrane was then incubated with secondary antibodies (Goat anti-chicken IgY HRP conjugated) (Abcam, Cambridge, UK) diluted to 1:1000 in TBST for 2 h at 37 °C. At last, the DAB-HRP color development kit (Beyotime, China) was used to determine the immune complexes.

### Generation and differentiation of DCs isolated from chicken spleen

Two-week-old healthy chickens were slaughtered and spleens were removed in an aseptic environment. Spleen tissues were macerated by frosted slides in PBS. Nylon cell strainer having 70 μm pore size (BD Biosciences) was used to filter the tissues for homogeneous suspension and splenic mononuclear cells were separated after Ficoll density gradient centrifugation. Cells (1 × 10^6^ cells/mL) seeded to six-well plates in 1640 RPMI complete medium (Gibco, Grand Island, NY, USA) containing 10% chicken heat-inactivated serum (Gibco, Grand Island, NY, USA), 20 ng/ml GM-CSF (Abcam, Cambridge, UK), 20 ng/mL IL-4 (Kingfisher, Saint Paul, MN, USA), 1% penicillin/streptomycin and cultured at 37 °C and 5% CO_2_ for 7 days. Next, the supernatant of the medium was aspirated after 12 h and replaced with fresh complete medium. At the 4th day, one-half of the original medium volume was removed and changed with fresh medium. At the 6th day, cells were incubated with rEaGAPDH (30 μg/mL), LPS (2 μg/mL; positive control; Sigma-Aldrich), pET-32a protein (30 μg/mL; negative vector control) or PBS (negative buffer control) for 24 h. At the 7th day, adherent cells were then harvested by gentle scraping with a cell scraper for downstream experiments. The cell cultures were photographed on 1st, 4th and 7th day of culture with a digital camera (Nikon, Tokyo, Japan) on an inverted microscope (Olympus, Japan) at 400× magnification.

### Fluorescence microscopy and laser confocal microscopy

Immunofluorescence assay was performed to test the interaction of rEaGAPDH protein with chicken DCs. In brief, the rEaGAPDH, pET-32a protein, or PBS pulsed DCs (1 × 10^5^) were settled on glass coverslips and fixed with 4% paraformaldehyde followed by the cells incubation in blocking buffer (PBS containing 5% BSA) for 1 h and subsequent incubation with primary antibody (sera against rEaGAPDH or normal rat-sera) for 1 h at 37 °C. After rinsing 3 times in PBS, the secondary antibody (chicken anti-rat IgG labelled with Cy3; Beyotime, Haimen, Jiangsu, China) was added and incubated for 1 h at 37 °C. Cells were then subjected to 40, 6-diamidino-2-phenylindole (DAPI), Sigma, USA) for 10 min and observed by a confocal laser scanning microscope (PerkinElmer, USA).

### Flow cytometric analysis

Cell-surface profiling of chicken DCs was carried-out by flow cytometry. The rEaGAPDH, LPS, pET-32a, or PBS treated DCs were adjusted at the rate of 1 × 10^6^ cells/mL. Double-color expression antibodies MHCII-FITC/CD1.1-PerCP-Cy5.5, MHCII-FITC/CD11c-PE, MHCII-FITC/CD80-APC, and MHCII-FITC/CD86-PE-Cy7 (Southern Biotech, USA) were used for the staining of isolated cells. The MHCII-FITC was served as a reference molecule. After 30 min of incubation with antibodies at 4 °C, the cells were evaluated by flow cytometry (BD Biosciences, San Jose, CA, USA) and were gated on MHCII^+^ expression.

### cDNA synthesis and relative qRT-PCR assessment

DCs treated with rEaGAPDH, LPS, pET-32a protein, or PBS were isolated as described in “Generation and differentiation of DCs isolated from chicken spleen” section. The cells were pelleted at 1500 rpm for 5 min at 4 °C followed by subsequent two washings with PBS. Total cellular RNA was purified using RNeasy plus kit (Qiagen) following the manufacturer's instructions. RNA concentrations were determined spectrophotometrically using Eppendorf Bio photometer Plus (Eppendorf AG, Hamburg, Germany) and all samples had an OD260/OD280 ratio of ~ 1.8–2.1. The RNA isolated from each cell type was reverse transcribed using the HiScript II Q RT SuperMix (Vazyme, Nanjing, China) kit, following the protocol provided by the manufacturer. The reaction volume (16 μL) containing 1 μg total RNA, 4 μl 4× gDNA wiper mix, and RNase-free water was thoroughly mixed in RNase-free PCR tubes. After incubation at 42 ℃ for 2 min, the mixture was supplemented with 4 μL of 5× HiScript II Select qRT SuperMix II (containing buffer, dNTPs, HiScript II reverse transcriptase, and RNase inhibitor). Thereafter, reverse transcription reactions were carried out at 50 °C for 15 min and 85 °C for 2 min using a thermocycler (BioRad, Hercules, CA, USA). Isolated cDNA was stored at − 80 °C until further use.

For qRT-PCR, forward and reverse primers were designed to amplify the fragments of target genes (Additional file 1). Quantitative amplifications were performed in ABI 7500 Fast Real-time PCR System (Applied Bio system, USA) using ChamQTM SYBR^®^ QRT-PCR master mix Kit (Vazyme, Nanjing, China). PCR was set up in microplates using a final volume of 20 μL containing 10 μL 2× SYBR QRT-PCR Master Mix, 0.5 μL each sense and antisense primer (10 μM), 0.5 μL 50× ROX reference dye-I, 1 μL cDNA and 7.5 μL DNA/RNA free deionized water. Amplification was performed according to the manufacturer's cycling protocol and done in triplicate. Amplification conditions were as follows: one cycle at 95 °C for 30 s, 40 cycles at 95 °C for 10 s, 60 °C for 30 s, and one extension cycle at 95 °C for 15 s, 60 °C for 60 s and 95 °C for 15 s. The data from the qPCR were analyzed using the threshold cycle (2−ΔΔCT) method [29]. The values obtained for target gene expression were normalized to those for β-actin expression.

### Cell proliferation assay

T cells from chicken spleens were isolated with nylon wool column as described previously [21]. Briefly, 10 mL plastic syringes were loaded with 0.1 g of nylon fibers and then autoclaved for sterility. Before use, columns were equilibrated by washing with 20 mL RPMI 1640 and were incubated for 30 min in 5% CO_2_ at 37 °C. Chicken spleen cells were washed with Hanks balanced salt solution. After lysis of red blood cells using RBC lysis buffer (BD Pharmingen, Franklin Lakes, NJ, USA), cells (2 × 10^8^) subjected to nylon wool purification were resuspended in 2 mL of warm RPMI 1640, loaded onto the column, and washed with 2 mL warm RPMI 1640. The column was sealed and incubated at 37 °C, 5% CO_2_ for 45 min. Non-adherent cells (mainly T lymphocytes) were eluted with 10 mL of RPMI 1640 preheated to 37 °C. T cell proliferation was tested at different DCs to T cells ratios (1:1, 1: 5 or 1:10) using cell counting kit-8 (CCK-8) assay (Beyotime Biotechnology, China). Cells were incubated in 96-well microtiter plates and stimulated with ConA (10 µg/mL) in 100 μL volumes in triplicate wells at 37 °C for 72 h. After incubation of CCK-8 solution (10 µL) for 6 h, cells were evaluated using OD450 value measured by spectrophotometer (Bio-Rad, USA).

### DC-mediated activation of CD3^+^/CD4^+^ T cells

rEaGAPDH, LPS, pET-32a protein or PBS treated DCs and T cells (1:5 DCs to T cell ratio) were co-incubated in six-well culture plates (1 × 10^6^ cells/mL). Cells were incubated for 7 days at 37 °C under 5% CO_2_ in the presence of RPMI medium (Gibco, Grand Island, NY, USA) containing 10% chicken serum (Gibco, Carlsbad, CA, USA; product code: 16110082), 15 ng/mL chicken IL-2 (Kingfisher, Saint Paul, MN, USA) and 1% penicillin/streptomycin. Seven days later, cells were collected and stained with FITC labelled CD3 and PE labelled CD4 antibodies (Southern Biotech, USA). Cell suspensions were finally assessed via flow cytometry (BD Biosciences, San Jose, CA, USA).

### Assessment of cytokines level by ELISA

The level of cytokines was evaluated using commercially available ELISA kits (Nanjing JinYibai Biotechnology, China) following the manufacturer's instructions. IL-10, IL-12, IFN-γ, and TGF-β cytokines were measured from the supernatants of rEaGAPDH, LPS, pET-32a protein, or PBS pulsed DCs. In addition, IL-4 and IFN-γ cytokines from supernatants of co-incubated DCs with T cells were also assayed.

### Cell viability assay

Annexin V-FITC apoptosis detection kit (Beyotime, China) was used to evaluate the percentage of live and apoptotic cells following kit instructions. In brief, cells were resuspended in binding buffer in the presence of annexin-V FITC and propidium iodide. Following incubation of 15 min, the stained cells were evaluated using flow cytometer (BD Biosciences, San Jose, CA, USA).

### Results analysis

All the statistical analyses were performed using GraphPad Prism (GraphPad software, Inc., CA, US) and statistical significance was calculated with one-way analysis of variance (ANOVA). Statistical data are presented as mean ± SEM of at least 3 independent biological replicates. *p ≤ 0.05; **p < 0.01; ***p < 0.001; ****p < 0.0001 denote a statistically significant difference and ‘ns’ denotes non-significant differences.

## Results

### Identification of rEaGAPDH protein by SDS-PAGE and western blotting

**Figure 1:**
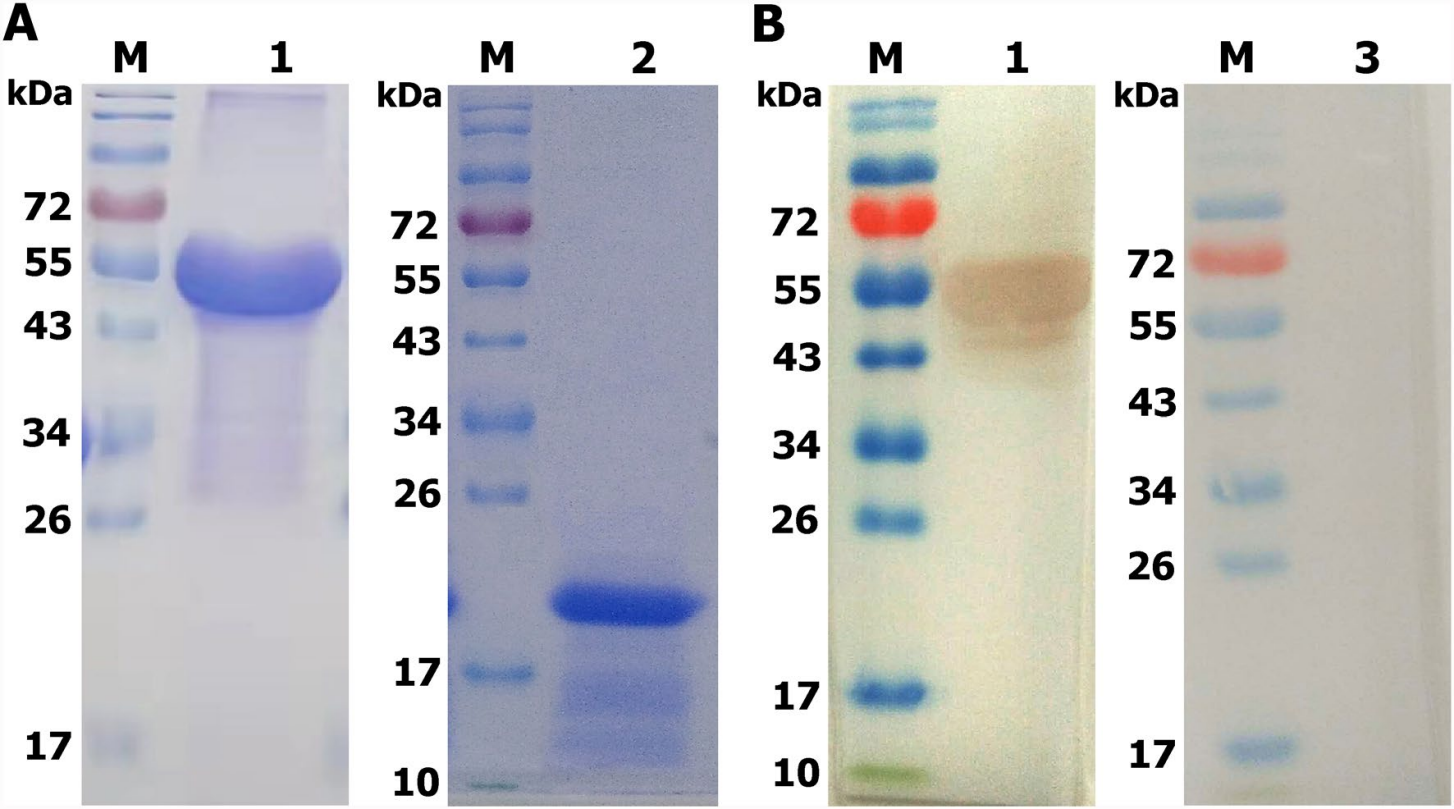
**Purification and immuno-blot analysis of rEaGAPDH protein. **M: molecular weight standard protein Marker. **A** Expression of purified rEaGAPDH protein (Lane 1) and pET-32a protein (Lane 2) resolved on SDS-PAGE. **B** M: molecular weight standard protein Marker; Lane 1: Purified rEaGAPDH was transferred to membrane and probed with serum from SD rats immunized with rEaGAPDH protein; Lane 2: Membrane probed with normal rat sera as control.

The detection of the purified rEaGAPDH protein band was confirmed through SDS-PAGE (Figure 1A). In addition, western blot analysis showed that a protein band of approximately 54 kDa strongly interacted with anti-rat rEaGAPDH antibodies (Figure 1B). However, the protein band was not detected from the sera of unimmunized rats. Results indicated that GAPDH was the immunogenic molecule of *E. acervulina* that can produce antibodies in the immune cells of rat host.

### Interaction of rEaGAPDH with chSPDCs

The interaction and internalisation of rEaGAPDH by DCs was analysed via immunofluorescence assay. rEaGAPDH-pulsed DCs were stained with anti-rEaGAPDH antibodies and detected by Cy3-conjugated secondary antibodies. Results indicated that red fluorescence on the surface of chSPDCs confirmed the interaction and internalisation of rEaGAPDH by DCs (Additional file 2). However, negative control groups did not show red fluorescence. In addition, nuclei of the cells were counterstained with DAPI and displayed blue fluorescence.

### rEaGAPDH-treated chSPDCs displayed morphological and phenotypical characteristics of mature DCs

The inverted microscope examination showed existence of various cell aggregates with few cytoplasmic projections on the 4th day of culture. Whereas, on day 7, the cells treated with rEaGAPDH or LPS exhibited typical morphology of mature DCs (i.e. increase in cell size, asymmetrical shape, multiple dendritic projections, and formation of cell clusters either semi-suspended or suspended in the medium) compared to the cells treated with PBS or pET-32a protein (Additional file 3).

**Figure 2:**
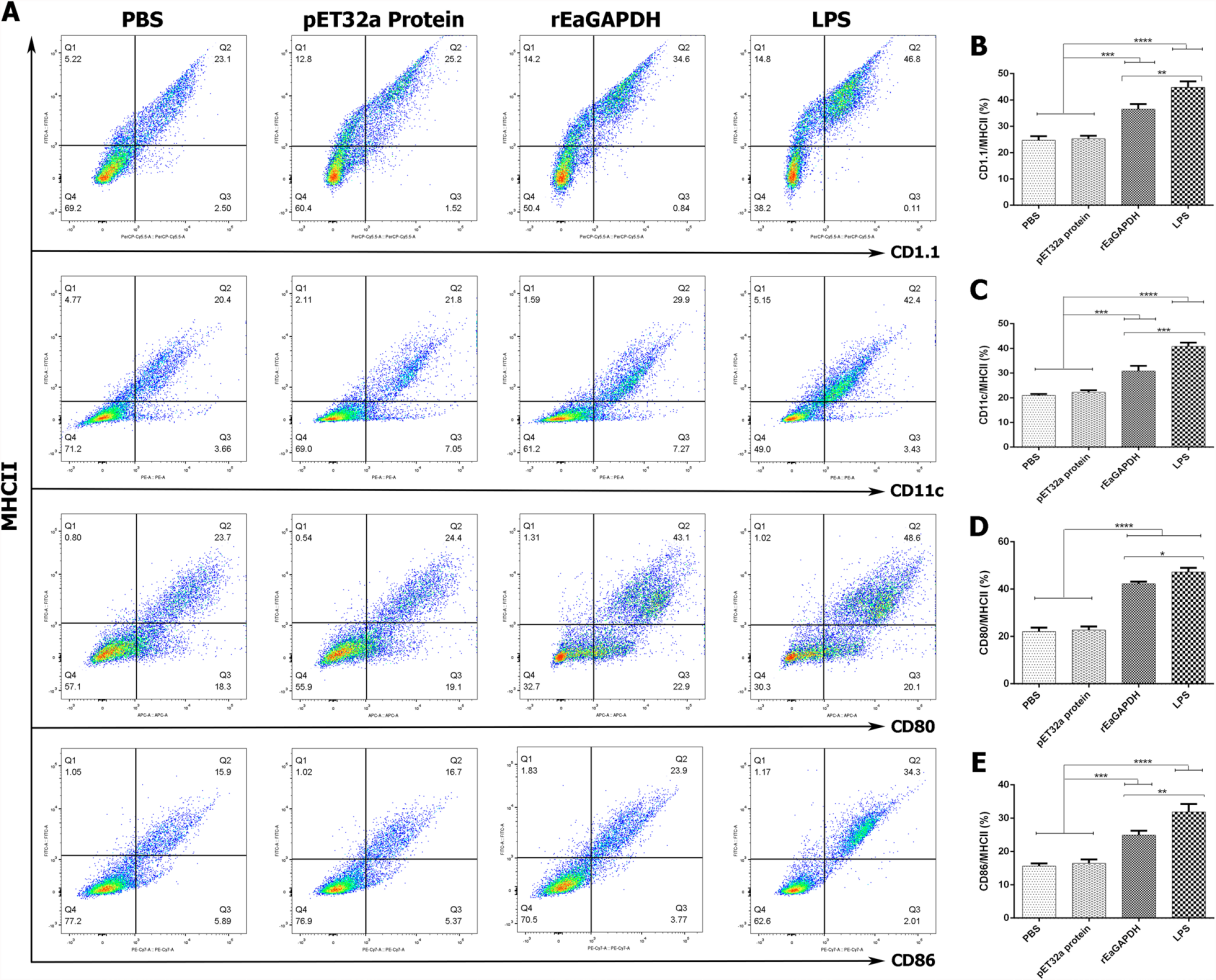
**Evaluation of phenotypic profile of mature DCs in response to rEaGAPDH.** Double-positive cell-surface markers in chicken DCs after incubated with rEaGAPDH, LPS, pET-32a protein or PBS were analysed by flow cytometry. **A** Dot plot percentage of MHCII/CD1.1, MHCII/CD11c, MHCII/CD80 and MHCII/CD86. Bar graphs showing percentage of **B** MHCII/CD1.1, **C** MHCII/CD11c, **D** MHCII/CD80 and **E** MHCII/CD86. Results presented here are from an independent experiment that is representative of three independent experiments (*p < 0.05, **p < 0.01, ***p < 0.001, and ****p < 0.0001).

To further evaluate whether rEaGAPDH-treated DCs can be identified by the phenotypic profile of mature DCs, the dual-colour expressions of markers for mature DCs and T cells costimulatory molecules were analysed by flow cytometry. Results indicated that, chSPDCs in response to rEaGAPDH and LPS (positive control) expressed higher levels of MHCII/CD1.1 (rEaGAPDH: p < 0.001, LPS: p < 0.0001), MHCII/CD11c (rEaGAPDH: p < 0.001, LPS: p < 0.0001), MHCII/CD80 (rEaGAPDH: p < 0.0001, LPS: p < 0.0001) and MHCII/CD86 (rEaGAPDH: p < 0.001, LPS: p < 0.0001) molecules than that of negative controls (Figure 2). Results suggest that rEaGAPDH induces phenotypic maturation of DCs that may stimulate T cells activation by costimulatory molecules.

### rEaGAPDH promoted the surface expressions of maturation biomarkers on chSPDCs

**Figure 3:**
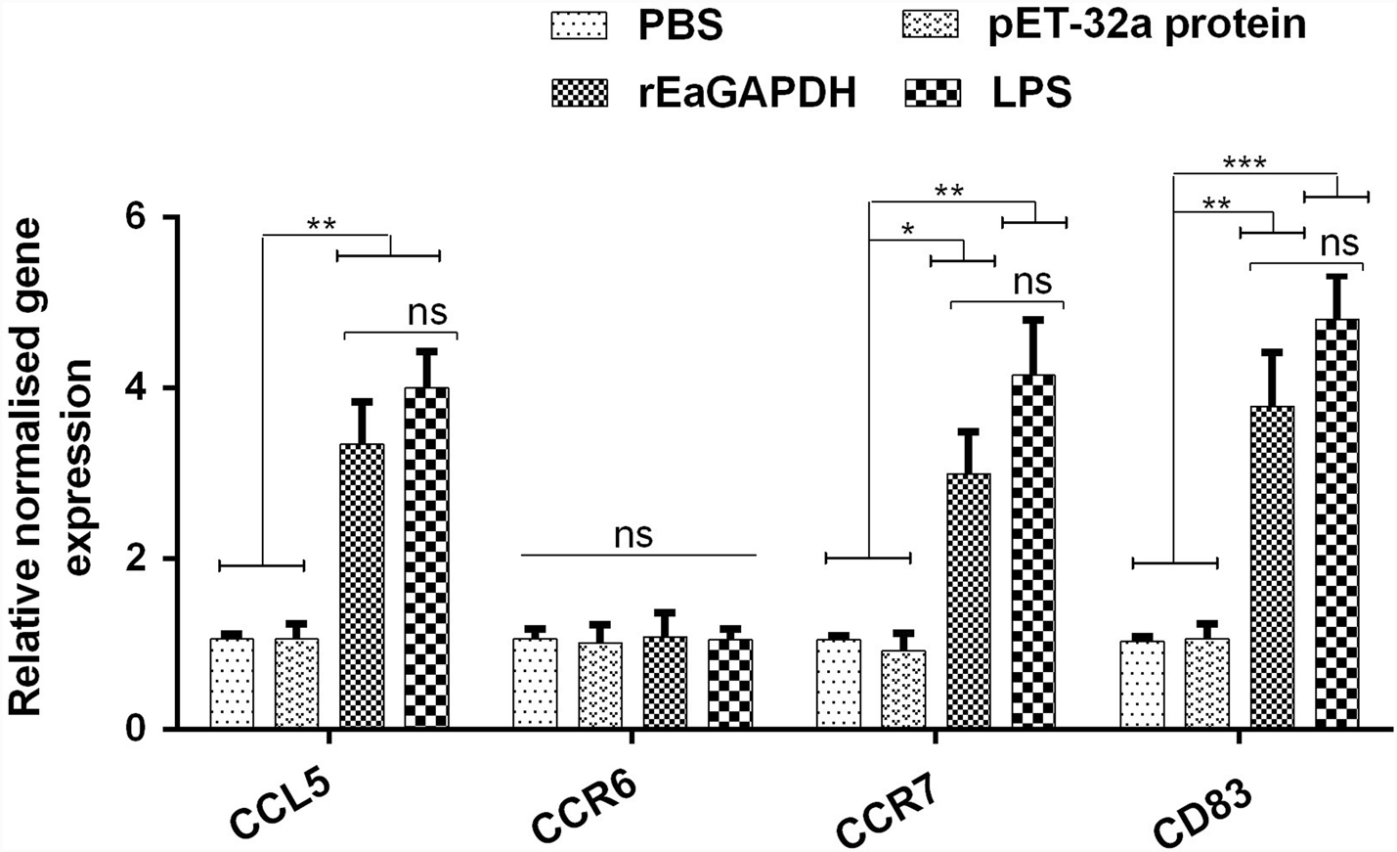
**The mRNA transcript profiles of DC maturation biomarkers in rEaGAPDH-treated DCs. **Cells were cultured with rEaGAPDH, LPS, pET-32a protein and PBS and assessed by qPCR. Symbolic data from one independent experiment with methodical triplicates representative of three independent experiments and the values presented here are the means ± SEM (*p < 0.05, **p < 0.01, ***p < 0.001, “ns” represent non-significant).

The mRNA transcripts of the biomarkers (CCL5, CCR6, CCR7, and CD83), commonly used for the identification of maturation state of DCs, were assessed via quantitative reverse transcription PCR (RT-qPCR). The expression levels of CCL5, CCR7, and CD83 were increased in chSPDCs under rEaGAPDH treatment (CCL5: p < 0.01, CCR7: p < 0.05, and CD83: p < 0.01) and LPS (CCL5: p < 0.01, CCR7: p < 0.01, and CD83: p < 0.001) whereas that of CCR6 were unchanged when compared with the negative controls (Figure 3). These outcomes clearly points that rEaGAPDH could stimulate the maturation of chicken DCs.

### rEaGAPDH modulated TLR and Wnt signalling pathways in chSPDCs

**Figure 4:**
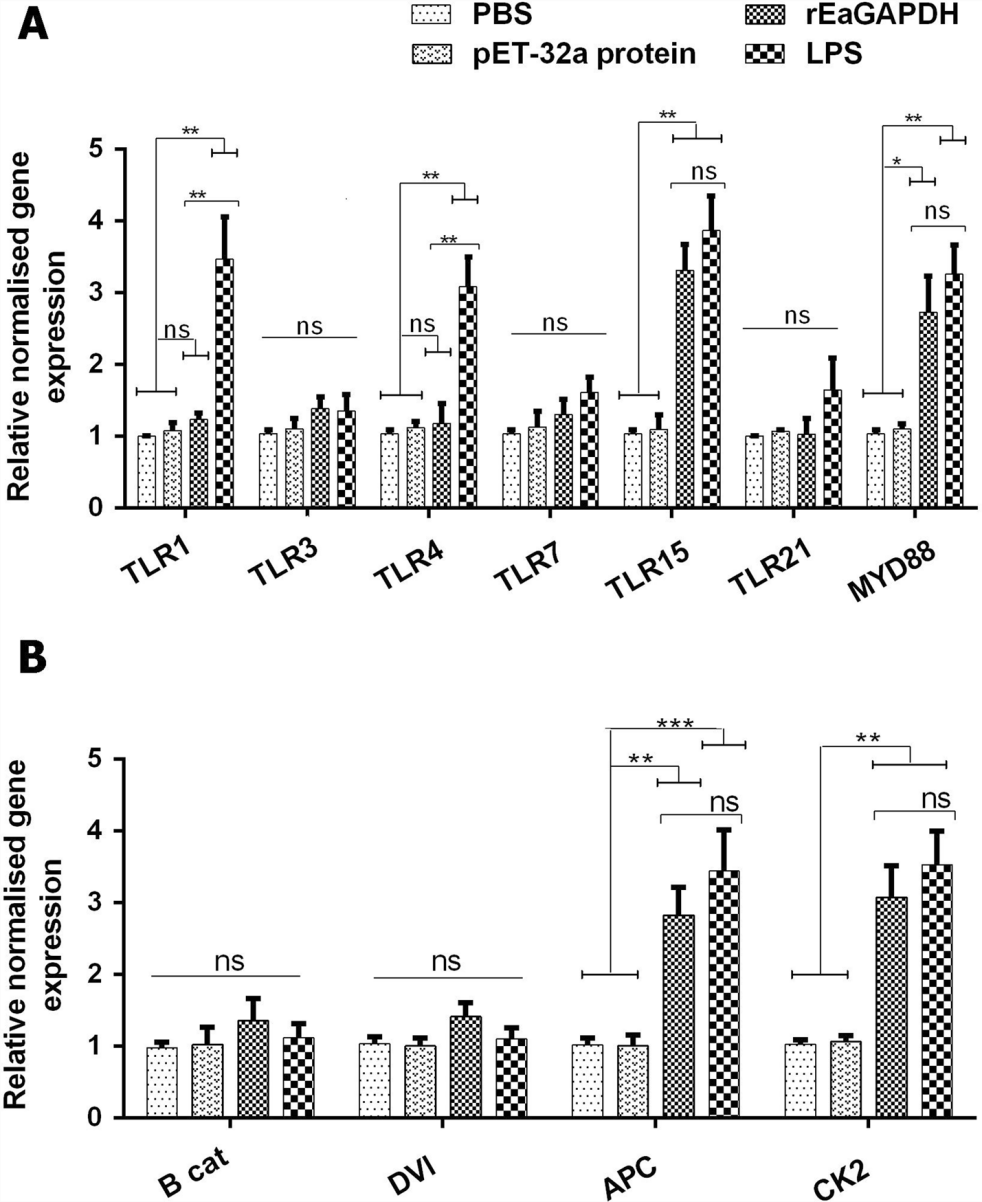
**The mRNA expressions of TLR signalling (A) and Wnt signalling (B) in rEaGAPDH-treated DCs.** DCs were treated with rEaGAPDH, LPS, pET-32a protein or PBS and evaluated by qPCR. Symbolic data from one independent experiment with methodical triplicates representative of three independent experiments and the values presented here are the means ± SEM (*p < 0.05, **p < 0.01, ***p < 0.001, “ns” represent non-significant).

The immunogenic maturation state of DCs was further confirmed by mRNA transcript profiles of TLR and Wnt signalling in DCs in response to rEaGAPDH. Among the TLR transcripts, our results showed higher expressions of TLR15 (p < 0.01), and MyD88 (p < 0.05) in rEaGAPDH-treated DCs, whereas*,* no differences were detected in TLR1, TLR3, TLR4, TLR7 and TLR21 compared with negative controls (Figure 4A). The increased expressions of TLR15 and MyD88 are a clear indication of the activation of TLR signalling in rEaGAPDH-pulsed DCs.

In addition, LPS-treated chSPDCs showed that transcript levels of TLR1 (p < 0.01), TLR4 (p < 0.01), TLR15 (p < 0.01) and MyD88 (p < 0.01) were upregulated, whereas TLR3, TLR7, and TLR21 did not significantly change compared to negative controls (Figure 4A).

Of the Wnt-β-catenin signalling genes, the increased expression levels of CK2 and APC were detected in rEaGAPDH-treated chSPDCs (CK2: p < 0.01, APC: p < 0.01) and LPS-treated chSPDCs (CK2: p < 0.01, APC: p < 0.001), while β-catenin and dishevelled (DVL) expressions were statistically unchanged compared to those of negative controls (Figure 4B). The increased expression of destruction complex genes (CK2 and APC) and maintained β-catenin and DVL levels suggest that rEaGAPDH may induce immunogenic maturation in chicken DCs.

### rEaGAPDH-treated DCs promoted the proliferation of naïve T cells

**Figure 5:**
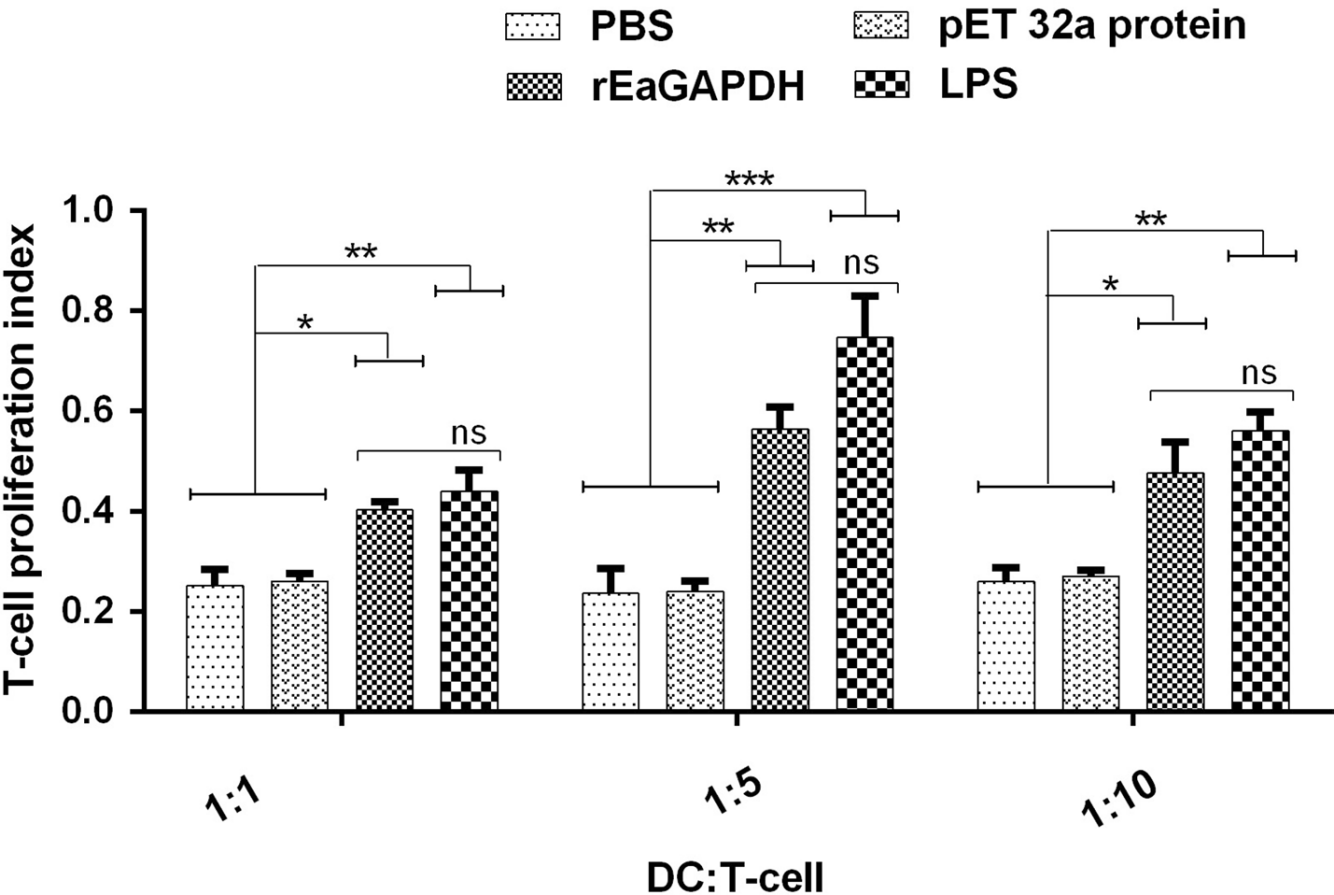
**Influence of rEaGAPDH on DC-dependent proliferation of T cells. **Cells were cultured with different ratios of DC to T cells (1:1, 1:5 and 1:10) and stimulated with ConA and rEaGAPDH, LPS, pET-32a protein or PBS. The proliferation assay was determined by CCK-8 incorporation after 72 h. The data are presented as the mean ± SD and representative of triplicate experiments (*p < 0.05, **p < 0.01 and ***p < 0.001, ns: non-significant).

The present study has evaluated the proliferation of naïve T cells in rEaGAPDH treated DC-T cell co-culture. Our findings showed that when compared with negative controls, the ability of DCs to stimulate naïve T cells to proliferate was enhanced after rEaGAPDH (p < 0.01) and LPS (p < 0.001) treatment (Figure 5). In addition, rEaGAPDH-treated or LPS-treated chSPDCs were stimulated T cells more efficiently when co-cultured in a ratio of 1:5 of DCs to T cells.

### rEaGAPDH modulated the release of cytokines in chSPDCs

**Figure 6:**
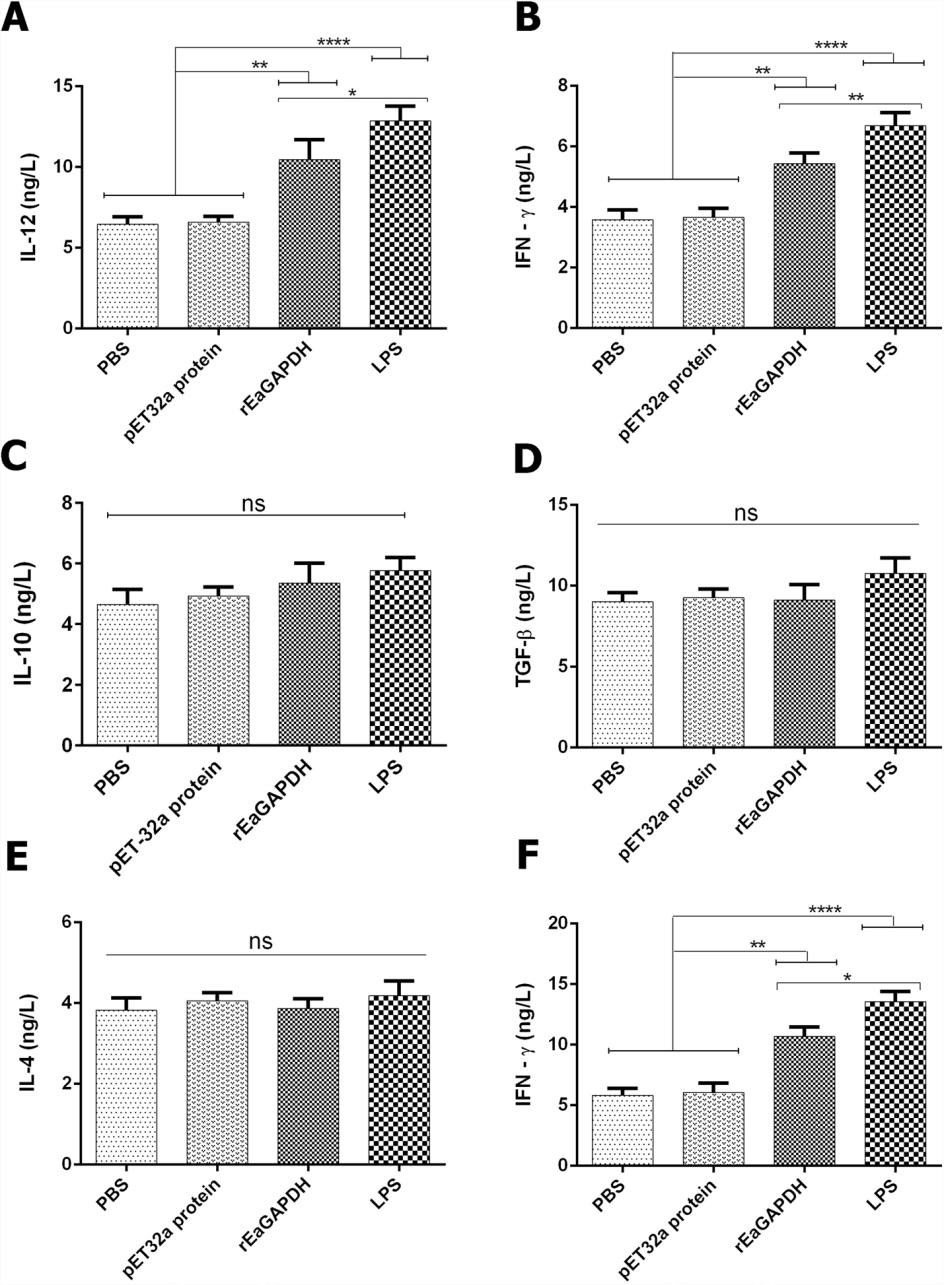
**Cytokine secretion pattern in chicken splenic-derived dendritic cells (chSPDCs) in repsonse to rEaGAPDH.** Cytokines levels in supernatants of chSPDCs and co-incubated DC-T-cell in response to rEaGAPDH, LPS, pET-32a protein, or PBS were quantified by ELISA kits. The production of cytokines **A** IL-12, **B** IFN‐γ, **C** IL-10 and **D** TGF-β in the supernatant of rEaGAPDH-pulsed chSPDCs. The cytokines **E** IL-4 and **F** IFN‐γ secreted by co-cultured chSPDCs with T-cell. The data were representative of three independent experiments and the values presented here were the means ± SEM (*p < 0.05, **p < 0.01, ****p < 0.0001 and “ns” represent non-significant).

The release of both pro-inflammatory (IL-12 and IFN-γ) and anti-inflammatory (IL-10 and TGF-β) cytokines from chSPDCs in response to rEaGAPDH was evaluated. Results indicated that the production of IL-12 and IFN-γ was increased in chSPDCs when treated with rEaGAPDH (IL-12: p < 0.01, IFN-γ: p < 0.01) and LPS (IL-12: p < 0.0001, IFN-γ: p < 0.0001) whereas those of IL-10 and TGF-β remained unchanged compared to negative controls (Figure 6A–D). These results suggest that rEaGAPDH favours the release of pro-inflammatory cytokines in chSPDCs.

### rEaGAPDH induced Th1 response in DCs-mediated T cells proliferation process

We further probed the secretion of Th1 (IFN-γ) and Th2 (IL-4) cytokines in DC-T cell co-culture. Our data showed that higher expression levels of IFN‐γ in T cells when co-incubated with rEaGAPDH-pulsed DCs (p < 0.01) and LPS-pulsed DCs (p < 0.0001) while IL-4 levels remained unchanged in comparison to negative controls (Figure 6E, F). These findings suggest that rEaGAPDH-pulsed DCs might drive T cells to favour Th1 response.

### rEaGAPDH-charged DCs and T cells co-culture efficiently stimulated allogenic CD4^+^ T cells

**Figure 7:**
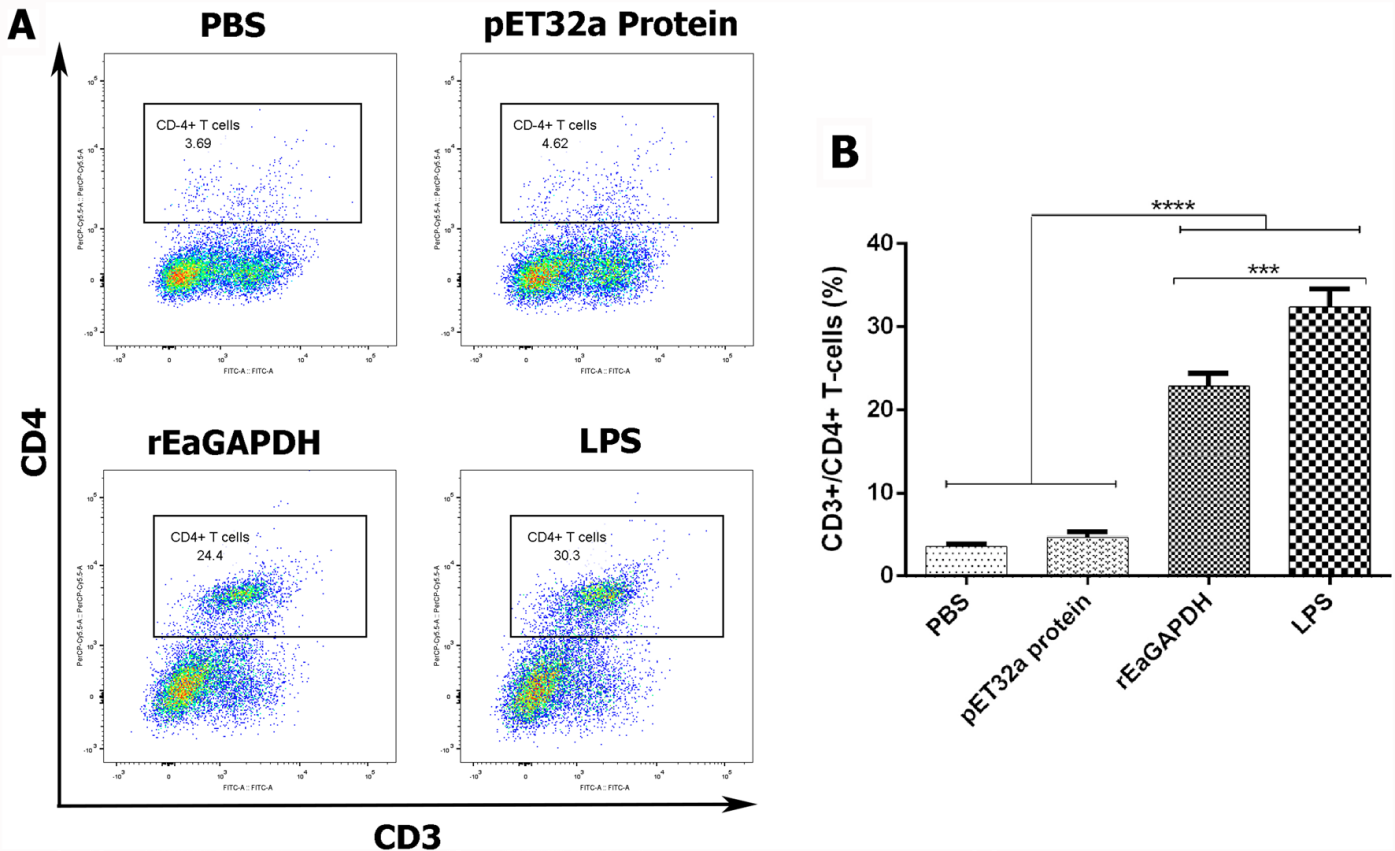
**Differentiation of naïve T cells into CD3**^**+**^**/CD4**^**+**^** T cells in response to rEaGAPDH.** rEaGAPDH, LPS, pET-32a protein or PBS treated DC were co-incubated with naïve T cells at the rate of 1:5 of chSPDCs to T cells at a final concentration of 1 × 10^6^ cells/ml and analysed by flow cytometry. **A** Dot plot percentage of CD3^+^/CD4^+^ T cells and **B** graph bar shows percentage of CD3^+^/CD4^+^ T cells. Data are presented as the mean ± SEM (n = 3) from triplicate experiments (***p < 0.001, ****p < 0.0001).

In order to evaluate the effect of rEaGAPDH-treated DCs on their capability to stimulate allogenic CD4^+^ T cells, the co-cultured DCs and T cells were double-stained with anti-CD3/CD4 T-cell markers. Flow cytometry results indicated that the expressions of CD3^+^/CD4^+^ T cells were significantly increased when T cells were co-incubated with rEaGAPDH-pulsed chSPDCs (p < 0.0001) and LPS-pulsed DCs (p < 0.0001) compared to negative controls (Figure 7). This suggests that DCs treated with rEaGAPDH can stimulate CD4^+^ T cells in a contact-dependent manner.

### rEaGAPDH did not change the viability of chSPDCs

**Figure 8:**
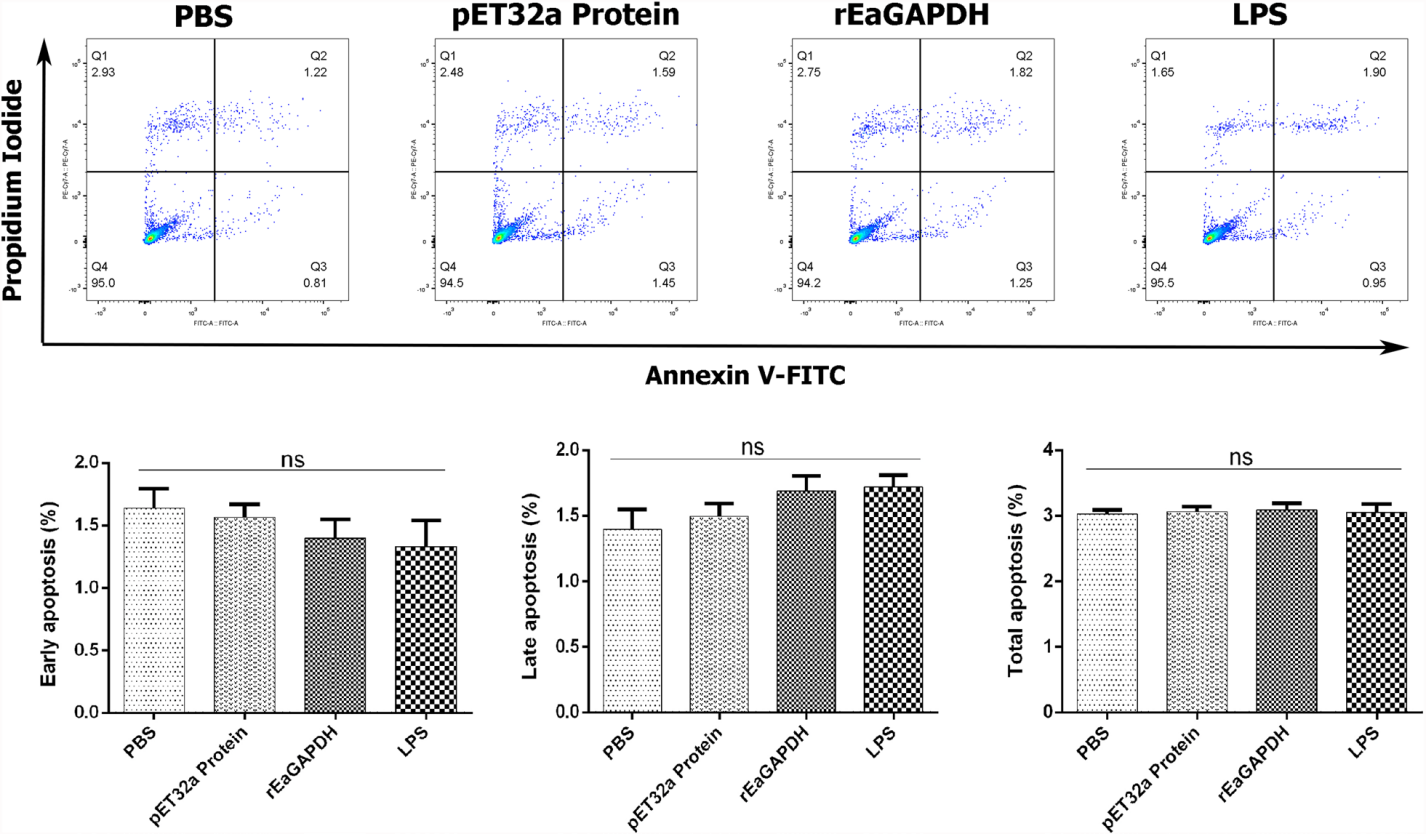
**Apoptosis assay of chicken splenic-derived dendritic cells (chSPDCs) in response rEaGAPDH.** **A** Apoptosis of chSPDCs was measured by flow cytometry after stained with annexin-V and PI. Bar graphs represent percentage of early stage apoptosis (**B**), late stage apoptosis (**C**) and total (early + late) apoptotic cells (**D**). Data are presented as the mean ± SEM (n = 3) from triplicate experiments. “ns” designate treatment groups are non-significantly differ to that of negative control groups.

To test whether rEaGAPDH induce programmed cell death in DCs, annexin V-FITC/PI assay was performed. Compared with negative controls, the percentage of apoptosis (early and late) in DCs treated with rEaGAPDH and LPS was unchanged (Figure 8). These results suggest that the viability of cells did not alter after rEaGAPDH exposure.

## Discussion

Cellular immunity is key for the control of intracellular parasites such as *Eimeria* [4, 20, 31]. In vaccination, the most critical step is the effective presentation of pathogen-derived antigens to T-cells and thereby elicit potent antigen-specific immune responses to pathogens [14]. Since DCs are the most efficient antigen-presenting cells and are crucial in inducing T-cell mediated immunity, they are believed to be the promising option for the evaluation of vaccine candidates [12, 24, 40]. Moreover, antigen-specific cross-talk between DCs and T cells is generally recognized as a major step in the development of adaptive immune responses [12]. In avian *Eimeria* parasites, the immunoproteomic analysis has recognized several common immunodominant antigens [28] and therefore is considered a useful breakthrough in exploring novel vaccine candidate. For instance, GAPDH is one of those antigens that are highly conserved in *E. tenella, E. maxima,* and *E. acervulina* [28], and potentially plays a crucial role in the invasion process [38]. The present study evaluates the *in-vitro* immunogenic roles of GAPDH from *E. acervulina* on the host DCs, the master regulators of the immune system, and confirms that rEaGAPDH is not only stimulant for the maturation of chicken DCs but also promotes their allostimulatory functions.

One of the hallmarks of mature DCs is to present antigens to CD4 T cells on MHC-II molecules via the provision of costimulatory signals [11]. It has been reported that the intensity of the signal that T cells receive from DCs is dependent both on the level of antigen-MHC and the level of costimulatory molecules [37]. We have revealed that rEaGAPDH-loaded DCs increased the expressions of MHCII, CD1.1, CD11c and costimulatory molecules CD80 and CD86. Our results are supported by an earlier study which indicated that chicken DCs modified their phenotypes after LPS treatment [44]. Moreover, we showed that expressions of biomarkers present on mature DCs (CCL5, CCR7, and CD83) were increased following rEaGAPDH treatment. These results are in accordance with the criteria used to identify the maturation state of DCs in chicken [25, 43, 44] as well as in mammalian species [16]. Thus, these results suggest that rEaGAPDH stimulates phenotypic maturation in chicken DCs.

Toll-like receptors (TLRs) play a crucial role in the innate immune system by recognizing the most diverse ligands from microbes [10]. A total of 10 different TLR genes and their ligands have been identified in chicken [18]. The binding of TLRs ligand on DCs leads to DC maturation. Activation of TLR signalling has been described to play a significant role in the host defence against pathogens in chicken [18]. In this study, among the analyzed TLR transcripts, only TLR15 expressions were upregulated in DCs in response to rEaGAPDH. Our results are consistent with earlier studies wherein expressions of TLR15 were increased in chicken immune cells following treatment of *E. tenella* [45] and Salmonella enterica serovar Typhimurium [15]. Activation of TLR by various ligands leads to the recruitment of downstream signalling pathways via MyD88, an essential adaptor molecule for all TLRs except TLR3 [13, 19, 32]. Previously, it has been reported that activation of chicken TLR15 is associated with adaptor protein MyD88 [5]. Similarly, we found increased expressions of MyD88 in DCs-treated with rEaGAPDH. These results suggest that rEaGAPDH might be the ligand for TLR15 and it could recruit MyD88 adaptor for the subsequent activation of nuclear factor- kappa B (NF-κB) signalling. However, activation of NF-κB and related signalling genes in response to rEaGAPDH in chicken DCs warrants further studies.

DCs can be matured either into an immunogenic or tolerogenic state [33, 46]. The signals necessary for the generation of tolerogenic DCs are important for understanding the nature of vaccine candidates. In this regard, the Wnt/β-catenin pathway plays a major role in DCs tolerization process [8]. This pathway gets activated through inhibition of the destruction complex (APC and CKs), which normally targets β-catenin for proteosomal degradation. However, DVL proteins inhibit the function of destruction complex causing the stabilization and accumulation of β-catenin [7, 33]. We showed increased transcript levels of APC and CK2 whereas those of β-catenin and DVL levels were unchanged in rEaGAPDH-loaded chSPDCs compared to negative controls. Our findings indicate that DCs matured in response to rEaGAPDH are in the immunogenic state.

DCs represent the interface between the foreign antigens and T cells, and they are the key players in the regulation of cell-mediated immune responses. T lymphocytes communicate with DCs in a highly competitive environment to obtain adequate stimulation of T cell receptor (TCR) for their activation and differentiation processes [37]. The potency of anticoccidial vaccines is considered to be evaluated in terms of T cells proliferation and differentiation [20]. Previously, the differential function of CD4^+^ T lymphocytes in offering resistance to primary and secondary coccidial infections has been documented [36]. In this study, the co-culture of rEaGAPDH-treated DCs and T cells revealed that proliferation of T cells and their differentiation into CD3^+^/CD4^+^ T cells were significantly increased. These findings suggest that the progressive process of T-cell differentiation and proliferation by random DC-T cell interactions following rEaGAPDH treatment results in the generation of terminally differentiated cells within the same responding clone which could promote the protective immune response against *Eimeria* infections. The exact mechanisms that trigger and maintain the sensitivity of naïve T cells during DC-T cell interaction in response to rEaGAPDH warrants further research.

Cytokines are crucial in the differentiation of naïve T cells into effector cells. However, the differentiation of Th1 or Th2 subtypes depends on the nature of infection (intracellular or extracellular) [17]. IL-12, the inducers of IFN-γ production, are produced by the mature DCs and promotes Th1 responses, which are essential for immunity to intracellular parasites including *Eimeria* [20]. Earlier studies have shown that chicken IFN-γ is capable of inducing anticoccidial immunity [30] and has an inhibitory effect on intracellular development of the parasite [26]. In the present study, the increased IL-12 and IFN-γ secretions in rEaGAPDH-treated chSPDCs were noted which might enhance Th1 type immune responses in the host. Interestingly, the interaction of rEaGAPDH-pulsed chSPDCs with T cells showed promoted secretion of IFN-γ, whereas the level of IL-4 secretion was unchanged, which further confirms the capability of rEaGAPDH to differentiate naïve T cells into Th1 subtypes. IL-10 cytokines are triggered by T regulatory cells (Treg) and mediate a suppressive role on host immune responses [3], however, TGF-β cytokines were considered to play regulatory roles in maintaining immune homeostasis [34]. We showed that the production of IL-10 and TGF-β in chSPDCs was not significantly changed. Our results suggest that rEaGAPDH is an important immunogenic molecule that might contribute to stimulating the host immune responses, predominantly Th1, against *Eimeria* species during host-parasite interactions. However, this study did not understand the regulatory mechanism of the host immune system for maintaining immune homeostasis and could be addressed in future studies.

In conclusion, rEaGAPDH takes part in the maturation of chicken DCs in an immunogenic state and enhances their ability to direct differentiation and proliferation of T cells. Moreover, rEaGAPDH could promote Th1 polarization by upregulating the production IL-12 and IFN-γ in chSPDCs and allogenic T cells. Our observations provide a new perspective on the function of this important molecule in stimulating the host immune responses and would contribute to the development of new DCs-based immunotherapeutic strategies to control coccidiosis. However, rEaGAPDH in combination with appropriate adjuvant to induce protective immunity needs to be further explored in the animal model.

## Supplementary information

**Additional file 1. Primers for RT-qPCR assays used in present study.**

**Additional file 2. Interaction and internalisation of rEaGAPDH by chicken splenic-derived DCs (chSPDCs).** chSPDCs were treated with rEaGAPDH, pET-32a protein or PBS and incubated with anti- rEaGAPDH, anti-pET-32a protein or negative rat IgG (as first antibody), followed by staining with Cy3-conjugated secondary antibody (red). Nuclei were counterstained with DAPI (blue) and visualized at confocal laser scanning microscopy at 1000× magnification. Merge are the overlaps of red and blue channels. No red fluorescence was observed in negative control groups.

**Additional file 3. Morphological observation of chicken splenic derived dendritic cells. ** Cells were cultured in the presence GM-CSF and IL-4 for 7 days and examined by Inverted microscope at 400x magnifications at day 1, 4 and 7. A1, A2, and A3: cells treated with PBS (negative buffer control); B1, B2 and B3: cells treated with pET-32a protein (negative vector control); C1, C2 and C3: cells treated with rEaGAPDH protein; D1, D2 and D3: cells were treated with LPS (positive control). The data are representative of at least three independent experiments.

## Data Availability

All data generated or analysed during this study are included within the article and its additional information files.

## Abbreviations

APC: adenomatous Polyposis Coli
BSA: bovine Serum Albumin
CCK-8: cell counting kit-8
chSPDCs: chicken splenic-derived dendritic cells
CK2: casein kinase 2
ConA: concanavalin A
DCs: dendritic cells
DVL: dishevelled
GM-CSF: granulocyte-macrophage colony-stimulating factor
MyD88: myeloid differentiation primary response gene 88
NF-κB: nuclear factor-kappa B
rEaGAPDH: recombinant glyceraldehyde-3-phosphate dehydrogenase of *Eimeria acervulina*
TLR: toll-like receptor
