# Supplementary material for: Glyceraldehyde-3-phosphate dehydrogenase from Eimeria acervulina modulates the functions of chicken dendritic cells to boost Th1 type immune response and stimulates autologous CD4+ T cells differentiation in-vitro
Source: Vet Res. 2020 Nov 17;51:138. doi: 10.1186/s13567-020-00864-z (PMC7672913; doi:10.1186/s13567-020-00864-z)
Supplement: Supplementary file 1 — Additional file 1. Primers for RT-qPCR assays used in present study. [file 13567_2020_864_MOESM1_ESM.docx]

| **Table. S1 Details of the primers for TLR signaling, Wnt signaling and DCs maturation markers.** | | | | | |
| --- | --- | --- | --- | --- | --- |
| **Target genes** | **Forward primer (5'-3')** | **Reverse primer (5'-3')** | **Temp (℃)** | **Amplicon length (bp)** | **NCBI reference ID** |
| **TLR1** | CTGCCAGTGGGCTGATTC | CGGTTGGATGCCAGGTTC | 60 | 183 | NM_001007488.4 |
| **TLR3** | TTGAATAGCCTTGATTTGTC | TTGTTCCTTGCAGTCCCT | 60 | 230 | NM_001011691.3 |
| **TLR4** | ACCCAACCACAGTAGCAT | CTTTCATCACCCATTCTT | 60 | 194 | NM_001030693.1 |
| **TLR7** | TGTGAATGAATGGGTGAT | CTAGAAGTCGTTGGTGGG | 60 | 229 | NM_001011688.2 |
| **TLR15** | CTTTGATGGGCTGTGGTATGT | GAAGGCATCGAAGGGCTTATT | 63 | 100 | MH143572.1 |
| **TLR21** | CTCACAGCACAATGCCTACA | GCAGTCCCAGCAAAGAGATAG | 62 | 84 | NM_001030558.1 |
| **CK2** | CGCTACATCCTCACCAACCG | AGCTTCACCATCGCCTCCC | 60 | 149 | XM_025145823.1 |
| **DVL** | GGGCAGATGTCGTTGACT | CCTGAGGTAGCCGTGTTT | 60 | 104 | XM_025142693.1 |
| **APC** | CTCATTCAACTGCCCAAAC | GCTATGTCGTGGCGTTTT | 60 | 100 | XM_001233410.5 |
| **β-catenin** | GGCAATCAAGAAAGTAAGC | AAGGTGGAGTCCTAAAGC | 60 | 192 | U82964.2 |
| **MYD88** | GTTTGATGCCTTCATCTGCT | CCTCCGACACCTTCTTTCTAT | 60 | 175 | EF011109.1 |
| **CCR6** | CGCTGTGGGCAGTGAATTA | GTACCGGTCCACACTGATAAAG | 62 | 134 | AM933593.1 |
| **CCR7** | GCTTTCAGCCTTCCTCTCAT | GACCTCCTTCTTCTCACACATC | 62 | 120 | HQ269806.1 |
| **CD83** | CTGGAGTTGGCGACAGAATAG | TTGAAAGGTGGTGTTGGAGAG | 62 | 101 | XM_418929.6 |
| **CCL5** | CCGTGTGCTGCTTCAACTAT | CTTCCTGGTGATGAACACAACT | 63 | 113 | NM_001045832.1 |
| **β-actin** | CTATCTGCCTTCACCACTTCTC | CTCAGAGAAAGCCAGCTTAGAG | 62 | 121 | X00182.1 |
