# Supplementary figures and images for: Glyceraldehyde-3-phosphate dehydrogenase from Eimeria acervulina modulates the functions of chicken dendritic cells to boost Th1 type immune response and stimulates autologous CD4+ T cells differentiation in-vitro
Source: Vet Res. 2020 Nov 17;51:138. doi: 10.1186/s13567-020-00864-z (PMC7672913; doi:10.1186/s13567-020-00864-z)

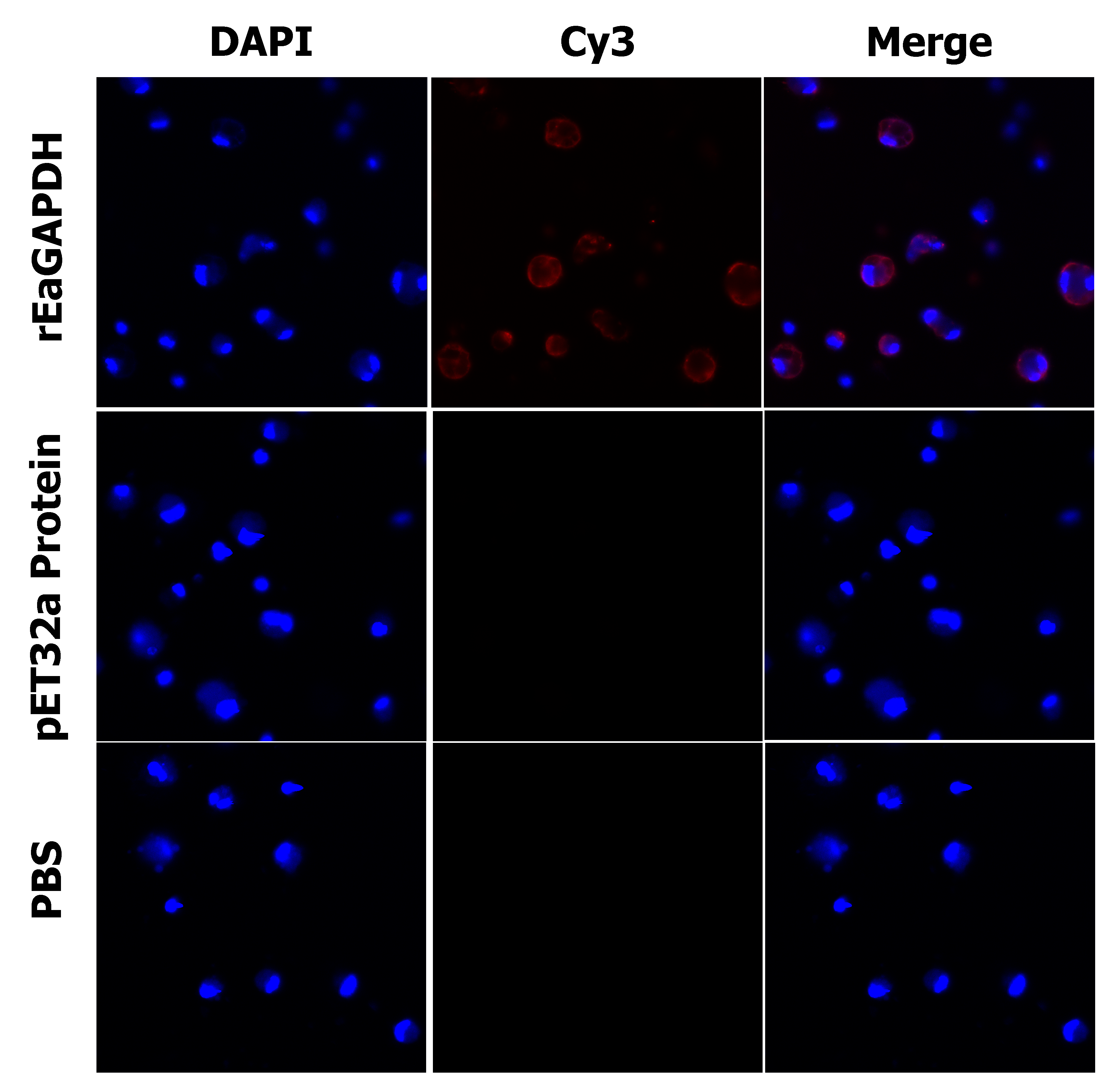

Supplement: Supplementary file 2 — Additional file 2. Interaction and internalisation of rEaGAPDH by chicken splenic-derived DCs (chSPDCs). chSPDCs were treated with rEaGAPDH, pET-32a protein or PBS and incubated with anti- rEaGAPDH, anti-pET-32a protein or negative rat IgG (as first antibody), followed by staining with Cy3-conjugated secondary antibody (red). Nuclei were counterstained with DAPI (blue) and visualized at confocal laser scanning microscopy at 1000× magnification. Merge are the overlaps of red and blue channels. No red fluorescence was observed in negative control groups. [file 13567_2020_864_MOESM2_ESM.tif]
